# Effects of Puberty Suppression and Sex Steroids on Weight, BMI, and Lipid Profiles in Danish Transgender Adolescents

**DOI:** 10.1210/clinem/dgaf549

**Published:** 2025-10-07

**Authors:** Kjersti Kvernebo Sunnergren, Pernille Badsberg Norup, Mette Ewers Haahr, Annamaria Giraldi, Anne Katrine Pagsberg, Peter Christiansen, Lise Aksglaede, Line Cleemann, Anders Juul, Katharina M Main

**Affiliations:** Department of Pediatrics, Göteborg Pediatric Growth Research Center (GP-GRC), Institute of Sciences, Sahlgrenska Academy, University of Gothenburg, Gothenburg 405 30, Sweden; Department of Pediatrics, Ryhov County Hospital, Region Jönköping County, 553 05 Jönköping, Sweden; Department of Growth and Reproduction, Copenhagen University Hospital—Rigshospitalet, 2100 Copenhagen, Denmark; International Centre for Research and Research Training in Endocrine Disruption of Male Reproduction and Child Health, Copenhagen University Hospital—Rigshospitalet, 2100 Copenhagen, Denmark; Sexological Clinic, Mental Health Center Copenhagen, Copenhagen University Hospital – Mental Health Services CPH, 2100 Copenhagen, Denmark; Department of Clinical Medicine, University of Copenhagen, 2100 Copenhagen, Denmark; Sexological Clinic, Mental Health Center Copenhagen, Copenhagen University Hospital – Mental Health Services CPH, 2100 Copenhagen, Denmark; Department of Clinical Medicine, University of Copenhagen, 2100 Copenhagen, Denmark; Department of Clinical Medicine, University of Copenhagen, 2100 Copenhagen, Denmark; Child and Adolescent Mental Health Centre, Mental Health Services, Capital Region of Denmark, 2900 Hellerup, Denmark; Department of Growth and Reproduction, Copenhagen University Hospital—Rigshospitalet, 2100 Copenhagen, Denmark; International Centre for Research and Research Training in Endocrine Disruption of Male Reproduction and Child Health, Copenhagen University Hospital—Rigshospitalet, 2100 Copenhagen, Denmark; Department of Growth and Reproduction, Copenhagen University Hospital—Rigshospitalet, 2100 Copenhagen, Denmark; International Centre for Research and Research Training in Endocrine Disruption of Male Reproduction and Child Health, Copenhagen University Hospital—Rigshospitalet, 2100 Copenhagen, Denmark; Department of Growth and Reproduction, Copenhagen University Hospital—Rigshospitalet, 2100 Copenhagen, Denmark; International Centre for Research and Research Training in Endocrine Disruption of Male Reproduction and Child Health, Copenhagen University Hospital—Rigshospitalet, 2100 Copenhagen, Denmark; Department of Growth and Reproduction, Copenhagen University Hospital—Rigshospitalet, 2100 Copenhagen, Denmark; International Centre for Research and Research Training in Endocrine Disruption of Male Reproduction and Child Health, Copenhagen University Hospital—Rigshospitalet, 2100 Copenhagen, Denmark; Department of Clinical Medicine, University of Copenhagen, 2100 Copenhagen, Denmark; Department of Growth and Reproduction, Copenhagen University Hospital—Rigshospitalet, 2100 Copenhagen, Denmark; International Centre for Research and Research Training in Endocrine Disruption of Male Reproduction and Child Health, Copenhagen University Hospital—Rigshospitalet, 2100 Copenhagen, Denmark; Department of Clinical Medicine, University of Copenhagen, 2100 Copenhagen, Denmark

**Keywords:** transgender, weight, body mass index, lipid profiles, gonadotropin-releasing hormone analog, sex steroids

## Abstract

**Context:**

Cardiovascular health of the transgender population receiving hormone therapy (HT) has been a concern.

**Objective:**

To investigate weight, body mass index (BMI), and lipid profiles in a national cohort of transgender adolescents starting HT before 18 years of age.

**Methods:**

In this observational study, 164 trans boys and 55 trans girls were followed longitudinally during HT. Gonadotropin-releasing hormone analog (GnRHa) was initiated either before or alongside sex steroid therapy. Anthropometry and lipid profiles were analyzed at the start of HT and at routine visits.

**Results:**

Before HT, overweight (BMI 1-2 standard deviation score [SDS]) and obesity (BMI ≥ 2 SDS) were found in 26.8% and 22.0% of trans boys, and in 5.7% and 5.7% of trans girls, respectively. BMI SDS correlated positively with total cholesterol, low-density lipoprotein (LDL), and triglycerides, and negatively with high-density lipoprotein (HDL). In trans boys and girls, high percentages had lipids above normal reference intervals; total cholesterol (12.5% and 6.1%), LDL (21.8% and 12.5%), and triglycerides (3.4% and 6.3%), and HDL below normal reference intervals (9.0% and 18.4%), respectively. During GnRHa monotherapy, there was a trend toward declining weight SDS, but BMI SDS and lipid profiles did not change consistently. After initiation of sex steroids, weight SDS, BMI SDS, and HDL decreased along with increased triglycerides in trans boys, and HDL increased in trans girls.

**Conclusion:**

Overweight, obesity, and dyslipidemia were common in transgender adolescents before HT initiation. BMI did not deteriorate, but dyslipidemia worsened slightly during sex steroid therapy in trans boys but not in trans girls.

Since 2016, hormone therapy (HT) has been available to transgender adolescents in a national tertiary center in Denmark. Gonadotropin-releasing hormone analog (GnRHa) may be offered from a minimum of Tanner stage 2, to block the endogenous pituitary-gonadal axis, and exogenous sex steroids, ie, testosterone or estradiol (17β-estradiol), are prescribed, to induce secondary sexual characteristics, usually after 15 years of age.

Body mass index (BMI) is a crude estimate of abdominal fat, but it is nevertheless associated with the amount of visceral fat mass, which in turn is a predictive factor for the development of metabolic complications in children and adolescents (1). High rates of overweight and obesity have been reported in transgender adolescents, especially in trans boys, before the start of HT (2-4). No significant changes in BMI standard deviation score (SDS) have been reported during GnRHa in neither trans boys nor trans girls (2, 5). Klaver et al reported an increase in BMI in both trans boys and girls during 2 years of GnRHa monotherapy and after the initiation of sex steroid therapy but did not disclose BMI changes in SDS. The study also found a higher prevalence of obesity in trans men and women compared to cis men and women at 22 years of age (6). In a larger study of children treated with GnRHa for precocious puberty, an increase in BMI SDS has been reported (7), and there have been some concerns about weight gain as a side effect of GnRHa treatment in transgender youth. In adults, both trans males and females have been shown to gain weight after the initiation of sex steroid therapy. Adult trans males tend to gain lean body mass and adult trans females predominantly gain fat body mass (8, 9).

Dyslipidemia, characterized by elevated levels of total cholesterol (TC), low-density lipoprotein cholesterol (LDL), and triglycerides (TG), alongside reduced levels of high-density lipoprotein cholesterol (HDL), is an important risk factor for cardiovascular disease (CVD) and is associated with premature atherosclerosis beginning in childhood (10). The prevalence of dyslipidemia in transgender adolescents before initiation of and during HT, is to our knowledge, not well studied. In one study, an increase in TC, LDL, and HDL was reported during GnRHa in both trans boys and girls (6). At 22 years of age and after initiating sex steroid therapy, HDL decreased, and TC, LDL, and TG increased in trans males. In trans females, only TG increased during the treatment period. Testosterone therapy in adult trans males has been associated with an increase in LDL and TG and a decrease in HDL (9, 11, 12). One meta-analysis and a prospective observational study also reported an increase in TC during testosterone therapy (13, 14). In adult trans females, reports on lipid profiles during feminizing HT are conflicting (11-14).

In summary, both visceral fat mass and dyslipidemia are risk factors for CVD. Since studies suggest an impaired risk profile for CVD (9, 11-16), CVD events (12, 17, 18) and CVD death (19) in adult transgender people receiving gender-affirming HT, understanding how this therapy affects the CVD risk profile in young transgender people is important. We hypothesized that HT would affect the CVD risk profile negatively, and we aimed to investigate weight, BMI, and lipid profiles before and during HT in a national cohort of transgender adolescents.

## Methods

### Study Population

This observational study reports data from a national Danish cohort of 219 transgender adolescents (164 trans boys and 55 trans girls) who started HT with GnRHa monotherapy or together with sex steroids, before 18 years of age, between January 1, 2016, and January 1, 2023. The cohort is part of the GenDa study, which is a retrospective study of all transgender adolescents in Denmark and has previously been described in detail (20, 21). A comprehensive medical, psychiatric, and psychosocial assessment was performed by a team of pediatric endocrinologists, child and adolescent psychiatrists, and child psychologists before HT was initiated. The assessment included an evaluation of the gender identity and the experience of gender and body dysphoria. All transgender adolescents met the criteria for HT of gender incongruence (21). Two trans boys who did not consent to participate in research were excluded before data extraction (20). A total of 103 (62.8%) trans boys and 42 (76.4%) trans girls started HT with GnRHa monotherapy. Of these, 19 (18.4%) trans boys and 14 (33.3%) trans girls had not yet started sex steroid therapy at the time of data extraction. Initiation of GnRHa and sex steroids was simultaneous in 61 (37.2%) trans boys and 13 (23.6%) trans girls. Among the cohort, 13 (5.9%) participants were treated for attention deficit hyperactivity disorder with central stimulants, 29 (13.2%) received antidepressants, 29 (13.2%) had been prescribed melatonin, and 4 (1.8%) were treated with antipsychotics. No individual was treated with statins, and no participants were diagnosed with an eating disorder.

### Hormone Therapy

The treatment protocol has previously been described in detail (20, 21). GnRHa (leuprorelin 11.25 mg subcutaneously or triptorelin 11.25 mg intramuscularly every twelfth week or 22.5 mg every 24 weeks) was used to suppress the ongoing pubertal development.

Testosterone or estradiol (17β-estradiol) therapy was initiated at a low dose and up-titrated every third to sixth month. Within a year of sex steroid therapy, the aim was to reach testosterone serum concentrations between 0 and +2 SD based on age-adjusted Danish male reference values. The goal of the estradiol therapy was to achieve estradiol serum concentrations of 200 to 400 pmol/L, corresponding to the mid-follicular phase in Danish females (22). The route of administration was determined based on individual preference and the physician’s recommendation.

Testosterone therapy was usually initiated by transdermal application of testosterone gel corresponding to 10 mg, gradually increasing to a daily dose of approximately 30 to 40 mg. When adult testosterone serum concentrations were reached, the administration route was usually changed to intramuscular injections of 1000 mg testosterone undecanoate, every twelfth week. Depending on the individual response, the interval of injections was adjusted to every tenth to eighteenth week. GnRHa monotherapy was stopped after reaching a serum testosterone concentration between 0 and +2 SD, according to the age-adjusted Danish male references values.

Estradiol therapy was usually initiated by transdermal application of patches or gel. Alternatively, tablets were offered. Treatment with patches was initiated at 12.5 or 25 µg/24 hours and gradually increased to 100 to 200 µg/24 hours. Gel was used when patches were not tolerated due to skin irritation. Treatment with estradiol tablets was initiated at a daily dose of 0.5 to 1 mg and increased to 4 to 8 mg. Treatment with GnRHa was continued during estradiol therapy.

### Data Collection

Data were collected at baseline, defined as closest to the start of any HT but no longer than 12 months before, and at routine visits every 3 to 6 months. Weight was measured to the closest 0.1 kg, wearing light clothing, using an electronic Floor scale (MPE 250K100PM, KERN & SOHN, Balingen, Germany). Height was measured to the closest 0.1 cm using a wall-mounted stadiometer (Harpenden Stadiometer, Holtain Ltd., Crymych, Britain). BMI was calculated as weight (kg)/(height [m])^2^. Weight SDS and BMI SDS were calculated according to the Danish reference data based on the sex assigned at birth (23). According to the World Health Organization (24), overweight was defined as BMI 1-2 SD and obesity ≥ 2 SD above the mean for age and sex assigned at birth. Nonfasting blood samples for analyses of TC, LDL, HDL, and TG were collected at routine visits. Dyslipidemia was defined according to normal reference intervals for nonfasting blood samples. For trans boys, the age of menarche was recorded. Bone age was measured when the height growth had not been completed. The difference between bone age and chronological age was calculated at baseline.

### Statistical Analyses

Microsoft Excel 2016 was used to draw the figures. Descriptive statistics, calculation of Pearson`s correlation coefficients (*r*), the paired *t* test and mixed model analyses were performed using the statistical software R, version 4.3.0 (http://cran.r-project.org/). Data for trans boys and girls were analyzed separately, except for the correlations between weight SDS/BMI SDS and the lipids, where data from trans boys and girls were merged. Mixed model analyses were conducted for both trans boys and trans girls at 12, 18, 24, 30, and 36 months after the start of GnRHa and sex steroid therapy, respectively. The results are presented as estimates [95% CI]. A *P* value of <.05 was considered statistically significant.

### Ethical Approval

The project was approved by the local ethics committee (H-18050607) and registered in the Capital Region of Denmark (P-2019-230), according to the general data protection regulation of the European Union. The Danish Patient Safety Authority (case no. 3-3013-3117/1), the Center for Health, Capital Region, and the Team for Medical Records Research (R-23014626) approved access to medical records, which included a waiver of consent. Data storage and analysis in Sweden were approved by the Swedish Ethical Review Authority (2023-02954-01).

The project is registered in ClinicalTrials.gov (NCT06573177).

## Results

### Baseline Characteristics

Data on age, anthropometry, and lipids at baseline are shown in Table 1. The prevalences of overweight (1-2 SDS) and obesity (≥2 SDS) were more pronounced in trans boys than in trans girls. Four (2.4%) trans boys had a weight SDS ≤ −2, and one (0.6%) trans boy had a BMI SDS ≤ −2. Four (7.5%) trans girls had weight and BMI SDS ≤ −2. Lipid concentrations outside the normal reference intervals were more often observed in trans boys than in trans girls, except for HDL, which was more often below the normal reference range in trans girls. The most striking findings were the high percentages of trans boys with elevated LDL (≥3 mmol/L) (21.8%) and trans girls with low HDL (≤1 mmol/L) (18.4%).

**Table 1.** Age, anthropometry and lipid profiles at the start of any hormone therapy in a national cohort of adolescents treated for gender incongruence

	Trans boys		Trans girls	
	n	Mean (SD)	n	Mean (SD)
Age, years	164	16.0 (1.5)	53	15.7 (1.7)
Weight, kg	164	63.6 (13.7)	53	61.3 (15.4)
Weight, SDS	164	0.8 (1.5)	53	−0.2 (1.2)
Height, cm	164	165.7 (7.0)	53	171.5 (9.7)
Height, SDS	164	−0.1 (1.1)	53	−0.2 (0.9)
BMI, kg/m^2^	164	23.1 (4.3)	53	20.6 (4.0)
BMI, SDS	164	1.0 (1.4)	53	−0.1 (1.2)

	n	%	n	%
Weight ≥ 2 SDS	29	17.7	4	7.5
BMI 1-2 SDS	44	26.8	3	5.7
BMI ≥2 SDS	36	22.0	3	5.7

	Trans boys		Trans girls	
	n	Mean (SD)	n	Mean (SD)
TC, mmol/L	144	4.0 (0.8)	49	3.8 (0.7)
LDL, mmol/L	147	2.4 (0.7)	48	2.2 (0.6)
HDL, mmol/L	145	1.4 (0.3)	49	1.3 (0.3)
Triglycerides, mmol/L	145	1.0 (0.5)	48	1.1 (0.6)

	n	%	n	%
TC ≥ 5.0 mmol/L	18	12.5	3	6.1
LDL ≥ 3.0 mmol/L	32	21.8	6	12.5
HDL ≤ 1.0 mmol/L	13	9.0	9	18.4
Triglycerides ≥ 2.0 mmol/L	5	3.4	3	6.3

Abbreviations: BMI, body mass index; HDL, high-density cholesterol; LDL, low-density cholesterol; TC, total cholesterol

Weight SDS correlated with LDL (*r* = 0.16, *P =* .028, n = 195), HDL (*r* = −0.24, *P <* .001, n = 194), and TG (*r* = 0.19, *P =* .008, n = 193). BMI SDS correlated with TC (*r* = 0.21, *P =* .004, n = 193), LDL (*r* = 0.28, *P <* .001, n = 195), HDL (*r* = −0.23, *P =* .002, n = 194), and TG (*r* = 0.22, *P =* .002, n = 193). No significant correlation was found between weight SDS and TC (*r* = 0.08, *P =* .265, n = 193). Correlations within the 2 groups had the same direction and the groups were therefore combined (data not shown).

In trans boys who were overweight, eight (18.2%) individuals were treated with antidepressants, one (2.3%) with a central stimulant, and one (2.3%) received an antipsychotic medication. Among trans boys with obesity, five (13.9%) individuals were treated with antidepressants, and one (2.7%) received a central stimulant. None of the trans girls with overweight or obesity were treated with any antidepressant, central stimulant, or antipsychotic.

Age at menarche was reported in 147/164 (89.6%) trans boys and menarche occurred at a mean age of 11.9 (SD 1.2) years. Six trans boys had not reached menarche at treatment start.

In trans boys (n = 85) and trans girls (n = 38), the difference (mean (SD)) between the bone age (years) and the chronological age (years) at baseline was 0.3 (1.1), *P =* .005, and 0.7 (1.6), *P =* .014, respectively.

### Anthropometrics During HT

**Figure 1.**
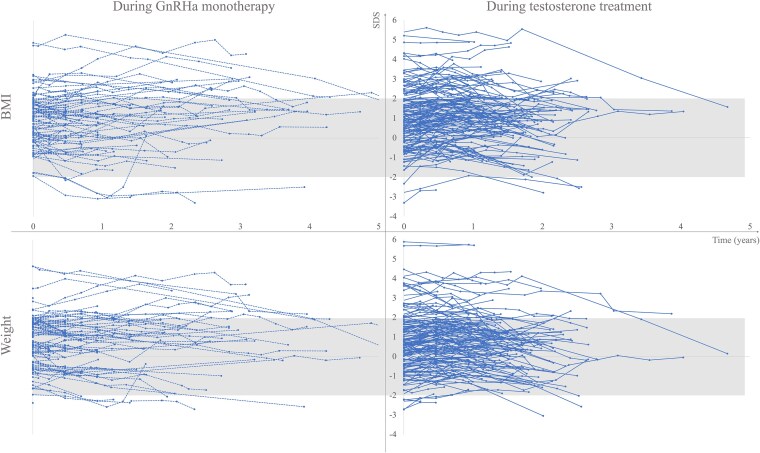
BMI SDS and weight SDS in trans boys during gonadotropin-releasing hormone analog (GnRHa) monotherapy and testosterone treatment. Note: The left panel depicts BMI and weight trajectories with dotted lines during GnRHa monotherapy (n = 103), and the right panel with solid lines during testosterone treatment (n = 145). The gray shaded areas depict BMI and weight between +/− 2 SDS. Data for those who started GnRHa and testosterone at the same time are shown during testosterone therapy. Abbreviations: BMI, body mass index; SDS, standard deviation score.

**Figure 2.**
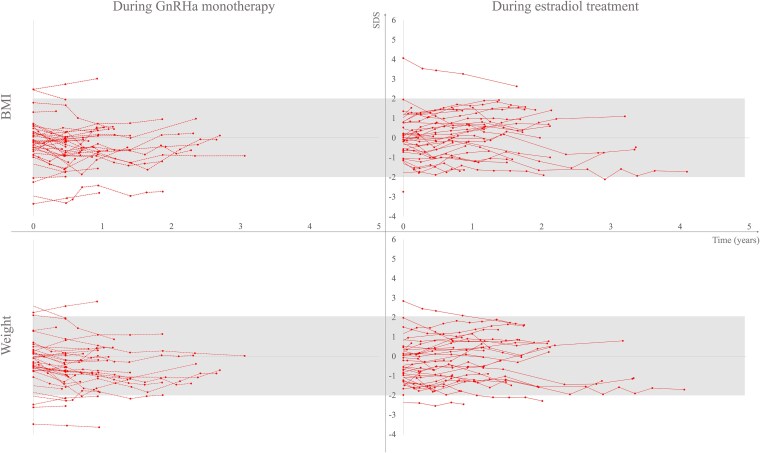
BMI SDS and weight SDS in trans girls during gonadotropin-releasing hormone analog (GnRHa) monotherapy and estradiol treatment. Note: The left panel depicts BMI and weight trajectories with dotted lines during GnRHa monotherapy (n = 43), and the right panel with solid lines during estradiol treatment (n = 41). The gray shaded areas depict BMI and weight between +/− 2 SDS. Data for those who started GnRHa and estradiol at the same time are shown during estradiol therapy. Abbreviations: BMI, body mass index; SDS, standard deviation score.

Figs. 1 and 2 depict the weight and BMI SDS during GnRHa monotherapy and after the addition of sex steroids. The trajectories were mainly within normal range for sex assigned at birth, and trajectories ≥2 SDS were more common in trans boys.

Changes in weight SDS and BMI SDS during GnRHa monotherapy are shown in Table S1 (25). A significant weight SDS reduction (estimate [95% CI]) was observed during the first year in trans boys (−0.2 [−0.3 to 0.0], *P =* .017 (n = 56)) and during the 2 first years in trans girls (−0.3 [−0.5 to −0.1], *P =* .010, n = 28 and −0.4 [−0.7 to −0.1], *P =* .013, n = 9), respectively. No significant change was observed in BMI SDS in any of the groups.

Table S2 (26) shows changes in weight SDS and BMI SDS after the initiation of sex steroids. After the start of testosterone therapy, weight SDS dropped significantly in trans boys after 2 years (−0.4 [−0.6 to −0.2], *P <* .001, n = 70) and 3 years (−0.7 [−1.2 to −0.2]), *P =* .006, n = 13) of treatment. No significant changes in weight SDS were observed for trans girls after the start of estradiol therapy. BMI SDS increased after 1 year of testosterone therapy in trans boys (0.2 [0.1 to 0.3], *P <* .001, n = 128) and decreased after 2 years of testosterone therapy (−0.2 [−0.4 to −0.0], *P =* .014, n = 70). In trans girls, BMI SDS increased after 1 year of estradiol therapy (0.2 [0.0 to 0.5], *P =* .048, n = 31).

### Lipids During HT

**Figure 3.**
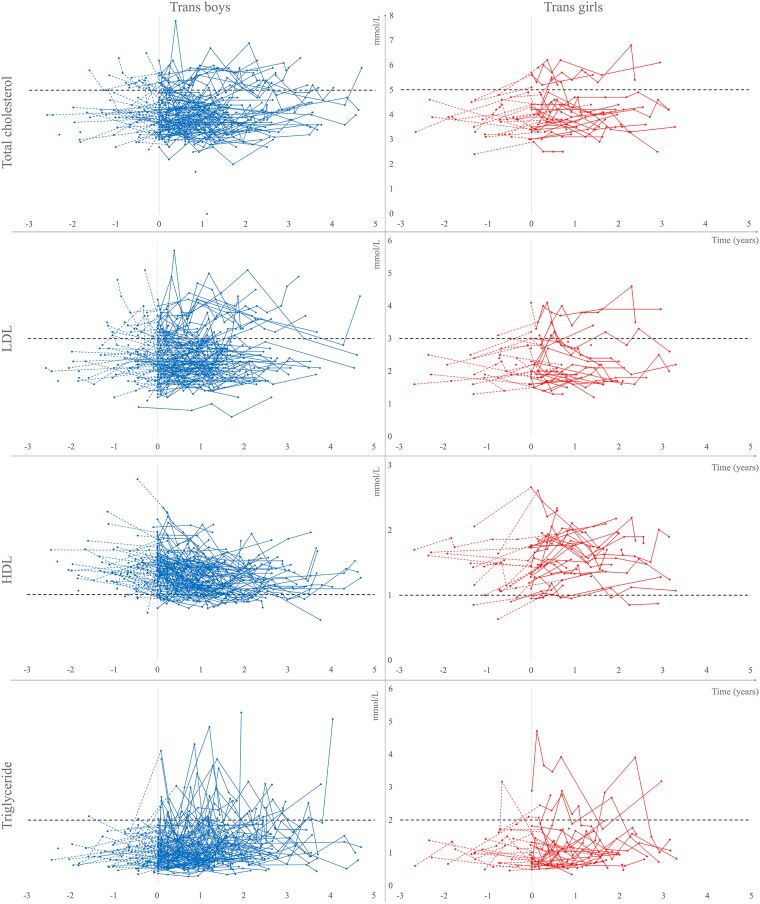
Lipids in trans boys and trans girls before and after initiation of sex steroid therapy. Note: TC (n = 144 and 41), LDL (n = 144 and 41), HDL (n = 144 and 41), and triglycerides (n = 144 and 37) are presented for trans boys (left panel) and trans girls (right panel) over time following initiation of sex steroid therapy (ie, testosterone or estradiol). The horizontal black dashed lines indicate the upper normal reference interval for TC, LDL, and triglycerides, and the lower normal reference interval for HDL. The colored dotted lines depict trajectories before sex steroid therapy (no therapy or GnRHa monotherapy), and the solid lines depict trajectories during sex steroid therapy. Abbreviations: GnRHa, gonadotropin-releasing hormone analog; HDL, high-density cholesterol; LDL, low-density cholesterol; TC, total cholesterol.

In Fig. 3, lipid concentrations over time are shown for trans boys and trans girls before and after initiation of sex steroids.

After the initiation of GnRHa monotherapy, the lipid profiles did not change significantly over time in neither trans boys nor trans girls, except for an increase in TC in trans girls after 1 year (0.4 [0.1 to 0.7], *P =* .007, n = 11), Table S1.

After the start of sex steroid therapy, HDL decreased in trans boys (−0.1 [−0.2 to −0.1], *P <* .001, n = 104, after 1 year, −0.2 [−0.2 to −0.1], *P <* .001, n = 62 after 2 years, −0.2 [−0.3 to −0.1], *P <* .001, n = 25 after 3 years of treatment, respectively) and increased in trans girls (0.3 [0.1 to 0.4], *P <* .001, n = 28, after 1 year, 0.3 [0.2 to 0.5], *P <* .001, n = 19 after 2 years, 0.4 [0.0 to 0.7], *P =* .038, n = 7 after 3 years of treatment, respectively), Table S2. TG increased in trans boys (0.2 [0.1 to 0.4], *P =* .003, n = 104, after 1 year, 0.3 [0.2 to 0.5], *P <* .001, n = 62 after 2 years, 0.4 [0.2 to 0.6], *P =* .002, n = 26 after 3 years of treatment, respectively) but not in trans girls. No significant changes were observed in TC or LDL.

## Discussion

In this unique single-center national cohort, we found a high proportion of treatment-naïve transgender adolescents, especially trans boys, with overweight or obesity, along with dyslipidemia. No significant change was observed in BMI SDS in any of the groups during GnRHa monotherapy, nor was any consistent trend observed during treatment with sex steroids. The effects of sex steroids on the lipid profiles differed between the groups during the first 3 years of treatment; in trans girls, HDL increased, and in trans boys, HDL decreased, and TG increased.

### Baseline Anthropometrics and Lipid Profiles

The high percentage of treatment-naïve overweight or obese trans adolescents, especially trans boys, was in line with previous reports (2-4). For comparison, a large population-based study of Danish adolescents reported a higher prevalence of overweight and obesity in cis boys than girls (27). The prevalence of obesity was higher in both trans boys and girls in the present study compared with Danish cis adolescents, and the prevalence of overweight was higher only in trans boys. Adolescents suffering from psychiatric disorders are at high risk of obesity (28), and psychopharmaceuticals, used by some of the participants in this study, have been suggested to contribute to the high rates of overweight and obesity in transgender youths (3, 4). Another explanation for the high rates of overweight and obesity could be the high prevalence of binge eating and compensatory eating behaviors described among transgender youths (29). Although no one in the cohort was diagnosed with an eating disorder, binge eating disorder, which is commonly associated with obesity, often goes undetected (30). Slightly earlier pubertal onset has been found in Danish adolescents reporting symptoms of gender incongruence (31). The age at menarche was 1.4 years earlier than expected (32) in trans boys, and the bone age was significantly advanced in both trans boys and girls. We do not know whether the age at menarche is still declining in the general population. In early-maturing pubertal children, there is a risk that the BMI percentile-for-age and obesity is overestimated (33), and early maturation may contribute to the high percentage of BMI SDS above normal reference intervals or vice versa.

In a population-based Danish cohort of children aged 6 to 19 years, the overall prevalence of dyslipidemia was 6.4%, which is lower compared to the findings of this study (34). In the same study, the prevalence of dyslipidemia in a cohort of overweight or obese children and adolescents was reported to be 28%, which is comparable to the findings of this study. Except for TC and weight SDS, we found significant positive correlations between TC, LDL, and TG, and negative correlations between HDL and both weight and BMI SDS, as expected. In a Danish population study, no age- or gender-specific differences were found in any of the lipids after 14 years of age (35). However, Nielsen et al and Balder et al reported age- and gender-specific patterns of lipid secretion during childhood and adolescence (34, 36), and thus it is uncertain whether early maturation affected the prevalence of dyslipidemia in this study.

Although lipid profiles during HT have been studied in both transgender adults (11-14) and adolescents (6), the prevalence of dyslipidemia in treatment-naïve transgender teens is, to our knowledge, not well studied. The prevalence of obesity and dyslipidemia in both trans boys and girls in this cohort suggests that lifestyle interventions, such as emphasizing the importance of physical activity, may be beneficial for transgender adolescents seeking HT.

### Anthropometrics During HT

Although the data should be interpreted with caution due to the limited number of repeated samplings, this study indicates that treatment with GnRHa and sex steroids does not negatively affect weight or BMI SDS.

Although speculative, the significant reduction in weight SDS but not BMI SDS during GnRHa monotherapy may reflect that further pubertal development ceased, resulting in reduced growth of muscle mass as described during the first treatment year in trans boys and the first 3 years in trans girls by Boogers et al (37). Another explanation could be that to achieve top surgery, trans boys needed to keep BMI at a maximum of 27 kg/m^2^ (38). Klaver et al reported increasing BMI during GnRHa monotherapy in both trans boys and girls (6). Still, they did not report BMI SDS, which in our opinion, better describes changes, as the BMI trajectory naturally increases until 18 years of age.

During the addition of sex steroids, the BMI SDS trajectories did not follow a consistent trend in any of the 2 groups, despite the significant reduction in weight SDS during the second and third year in trans boys. Klaver et al reported increasing BMI during puberty blockers and subsequent sex steroid therapy, but they did not report changes in BMI SDS (6). Since overweight and obesity were common in trans boys, the reduction in weight SDS after 2 and 3 years of testosterone therapy may be a result of reduced fat mass as described by Boogers et al during the first year of testosterone therapy in trans boys (37). Despite the lack of a significant change in weight SDS after 1 year of sex steroid therapy, both trans boys and girls in this study had increasing BMI SDS, followed by decreasing BMI SDS in trans boys.

### Lipid Profiles During HT

Except for the increasing TC in trans girls, analyzed only after 1 year of GnRHa monotherapy due to the limited data available, the present study did not confirm data reported by Klaver et al (6), who reported increasing TC, LDL, and HDL during GnRHa monotherapy in trans adolescents.

After the addition of sex steroids, the present study showed consistent trajectories for the decrease of HDL and the increase of TG in trans boys and the increase of HDL in trans girls. The changes were small, and the clinical relevance remains uncertain. The increase in HDL in trans girls was consistent with the findings reported by Robinson et al (39) but not in line with a study by Klaver et al, who reported changes in lipid profiles from 15 to 22 years of age, during GnRHa monotherapy and subsequent addition of sex steroids (6). However, in young trans males, Klaver et al reported a decrease in HDL and an increase in TG, consistent with the findings of the present study. In the adult transgender population, studies have consistently described decreasing HDL in trans men receiving masculinizing HT (9, 11-16). Although increasing HDL has been reported during feminizing HT (16, 40), both decreasing (15) and stable (11, 13, 14) HDL concentrations have also been reported. The decrease in HDL found in trans boys is a matter of concern because a longitudinal study found that higher HDL during adolescence was associated with lower risk of CVD during middle adulthood, and that a decrease in HDL from early childhood to adolescence resulted in an increased risk of adult CVD (41). In contrast to the findings of the present study, Klaver et al reported increased TC and LDL concentrations in young trans males and increased TG concentration in young trans females. These discrepancies may be due to the limited follow-up period resulting in a limited number of repeated measurements in the present study. However, the findings of the present study are in line with the TG changes reported in adult transgender people on sex steroid therapy. An increase in TG has been reported in adult trans males during testosterone therapy (11, 13-16). In adult trans females, the results are conflicting. Both increasing (11, 16), decreasing (14), and stable TG (13) concentrations have been reported during estradiol therapy. Higher levels of TG during childhood are associated with CVD events before 60 years of age, especially when combined with youth smoking, and therefore, the increase in TG seen in trans boys is worrisome (42). Furthermore, increasing LDL has been reported in adult trans males receiving testosterone (9, 11-16). In adult trans females receiving estradiol, both decreasing LDL (12, 14-16), stable LDL (11), and inconsistent results (13) have been reported. An increase in TC has been reported in adult trans males during testosterone treatment (13-15) and a decrease has been reported in trans females during feminizing therapy (14, 15). In line with the results of the present study, some studies have reported no change in TC during sex steroid therapy in trans males (9, 11) or trans females (11, 13).

### Strengths and Limitations

One major strength of this study was that the results were based on a unique dataset from a national cohort of transgender youths, and that all the data were collected in a single-center setting. Another strength was that baseline data were available in the study.

The study had certain limitations. First, the short follow-up period, resulting in a limited number of repeated measurements. Second, the cohort was homogenous, mainly consisting of white Caucasians, and the results of the study may not be applicable in other populations. Third, the study was conducted in a clinical setting, and the occurrence of missing data was another limitation. Fourth, BMI and weight alone may not fully capture the effects of hormone therapy on lean body mass. Lastly, the lipid profiles were analyzed using nonfasting blood samples, possibly overestimating the TG levels.

## Conclusion

This study showed that overweight, obesity, and dyslipidemia were common in trans boys and girls before any HT was initiated. GnRHa monotherapy seems to be safe and does not negatively affect weight or BMI. During sex steroid therapy, lipid profiles deteriorated in trans boys with decreasing HDL and increasing TG. In trans girls, however, an increase in HDL was found. This study emphasizes the importance of lifestyle interventions, such as encouragement of physical activity and prevention of tobacco use, as part of the treatment program for transgender youths, even before any HT is initiated. The clinical significance of the changes found in the lipid profiles for the risk of a CVD event and early CVD death remains uncertain, but it is an essential question for further research.

## Data Availability

The dataset of this study is not publicly available but can be made available from the corresponding author on reasonable request including adherence to legal requirements for confidentiality and data transfer.

## Abbreviations

BMI: body mass index
CVD: cardiovascular disease
GnRHa: gonadotropin-releasing hormone agonist
HDL: high-density lipoprotein cholesterol
HT: hormone therapy
LDL: low-density lipoprotein cholesterol
SDS: standard deviation score
TC: total cholesterol
TG: triglycerides
